# Apparent Time Interval of Visual Stimuli Is Compressed during Fast Hand Movement

**DOI:** 10.1371/journal.pone.0124901

**Published:** 2015-04-08

**Authors:** Takumi Yokosaka, Scinob Kuroki, Shin’ya Nishida, Junji Watanabe

**Affiliations:** NTT Communication Science Laboratories, Nippon Telegraph and Telephone Corporation, Kanagawa, Japan; University of Melbourne, AUSTRALIA

## Abstract

The influence of body movements on visual time perception is receiving increased attention. Past studies showed apparent expansion of visual time before and after the execution of hand movements and apparent compression of visual time during the execution of eye movements. Here we examined whether the estimation of sub-second time intervals between visual events is expanded, compressed, or unaffected during the execution of hand movements. The results show that hand movements, at least the fast ones, reduced the apparent time interval between visual events. A control experiment indicated that the apparent time compression was not produced by the participants’ involuntary eye movements during the hand movements. These results, together with earlier findings, suggest hand movement can change apparent visual time either in a compressive way or in an expansive way, depending on the relative timing between the hand movement and visual stimulus.

## Introduction

Accurate estimation of time intervals is an essential sensory ability for dynamic interaction with the external environment through body movements [1]. Growing evidence indicates a relationship between body movements and the estimation of time. For example, the increase of perceived duration of a visual moving stimulus with stimulus motion speed [2–6] is weakened when the observer's eye or hand tracks the moving stimulus [7]. The compression of tactile duration after adaptation to tactile motion is canceled out by a voluntary hand motion [8]. These findings suggest that body movements make the estimations of time more veridical, but this is not a general rule. Many other studies have reported that body movements can make the estimations of time less veridical [9–16].

Some studies reported that the intervals of visual events presented before and after a voluntary manual movement are perceptually expanded [12, 16]. These studies suggest that the execution of hand movement expands the estimated time of a visual stimulus. Other studies reported that the apparent visual interval is perceptually compressed immediately before and during a saccadic movement or during a smooth pursuit eye movement [13, 14] and that the apparent tactile interval is compressed immediately before and during a hand movement [15]. These findings suggest that the estimated time is compressed during the execution of a body movement.

This study examined visual time estimation during the execution of a hand movement. In daily life, we often see something while moving a part of our body, but how we estimate visual time in such situations remains unknown. A visual time estimation and a hand movement is the stimulus-action combination with which Hagura et al. [16] and Park et al. [12] found time expansions, while a visual time estimation during an hand movement is the stimulus-action relative timing with which Morrone et al. [13] and Schütz and Morrone [14] found time compressions. Therefore, visual time estimation during a hand action is a critical condition for examining whether the effect of the action on time perception is determined by the combination of stimulus and action or by the relative timing between stimulus and action.

In our experiment, we examined whether the estimated interval marked by visual events is affected during the execution of hand movement. Our results show that hand movements, at least fast ones, compressed the apparent time interval between visual events. The exclusion of trials in which participants moved their eyes did not affect this apparent time compression, which excluded the possibility that the apparent time compression was produced by involuntary eye movements during hand movements. These results suggest that the visual time is apparently compressed during manual movements like it is during eye movements [13, 14], despite the visual time’s being apparently expanded before and after manual movements [12, 16].

## Results

**Fig 1:**
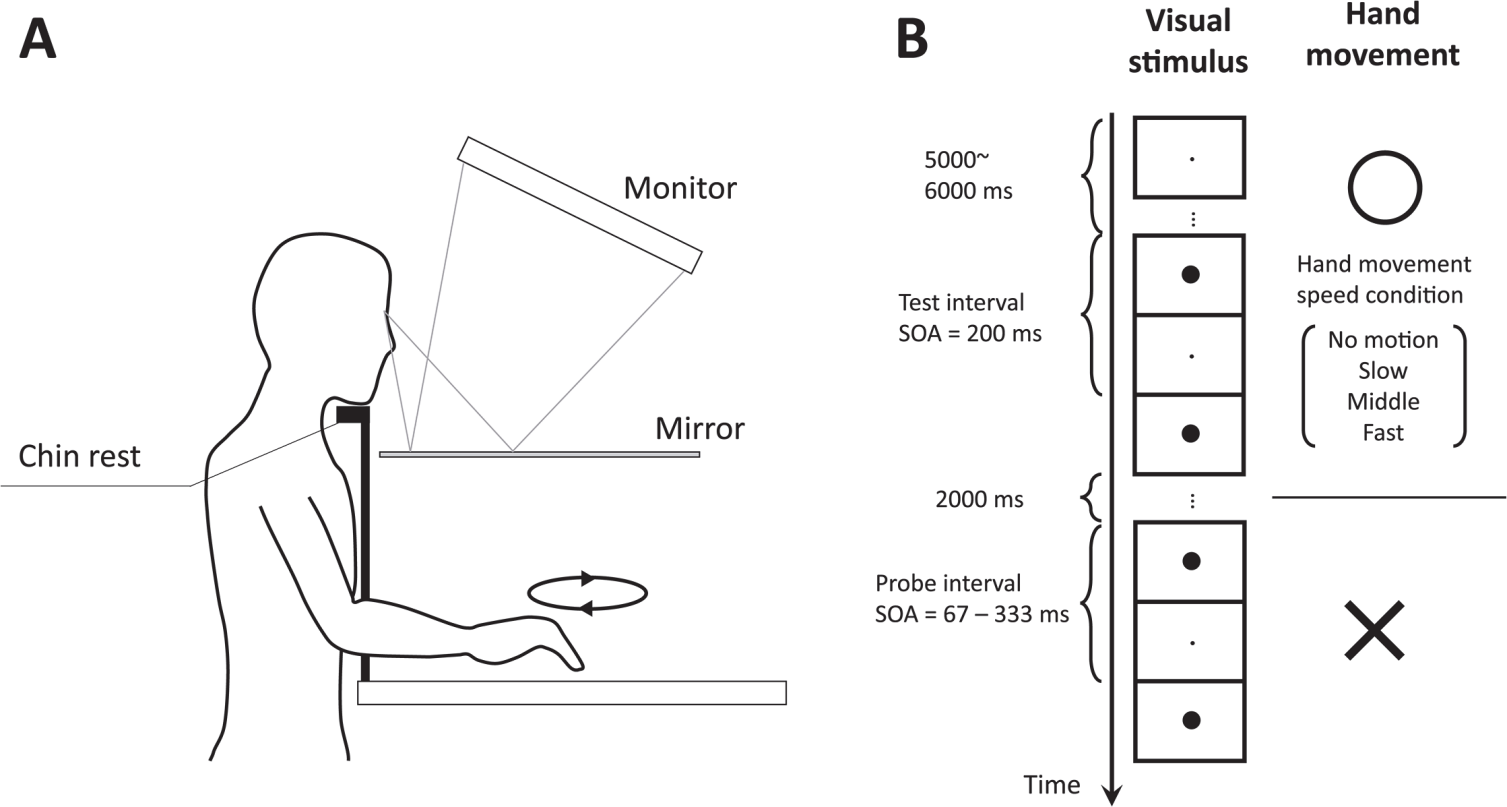
Schematic illustration of the experimental setup and time course. (A) Experimental setup. (B) Time course of a stimulus for the experimental conditions.

In each trial of our experiment, two pairs of visual stimuli were presented sequentially. The interval between the two stimuli of the first pair (test interval) was fixed at 200 ms; that between the two stimuli of the second one (probe interval) was varied. Participants moved their right hand circularly around the fixation point at a constant speed [zero (no motion), slow, intermediate, or fast] when observing the test interval (as shown in Fig 1A, a mirror prevented them from viewing their hands and arms) and immediately stopped the hand movement when observing the probe interval. They made a judgment as to the relative length of temporal intervals defined by the two visual flashes (Fig 1B).

The radii of the hand movements were 69.39 ± 9.31, 66.89 ± 11.85, and 69.31 ± 11.93 mm (mean ± SD across participants) in the slow, intermediate and fast movement conditions, respectively. A one-way repeated measures ANOVA indicated no significant main effect (F(2, 18) = 1.23, P = 0.33, *η* ^2^ = 0.01). In addition, the standard deviations of the radii for each participant in the slow, intermediate, and fast conditions were 10.61 ± 3.93, 9.07 ± 2.80, and 9.12 ± 3.03 mm (mean ± SD across participants), respectively. A one-way repeated measures ANOVA indicated no significant main effect (F(2, 18) = 1.72, P = 0.22, *η* ^2^ = 0.05). These results indicate that the radii of the hand movement were not different between three hand movement conditions.

**Fig 2:**
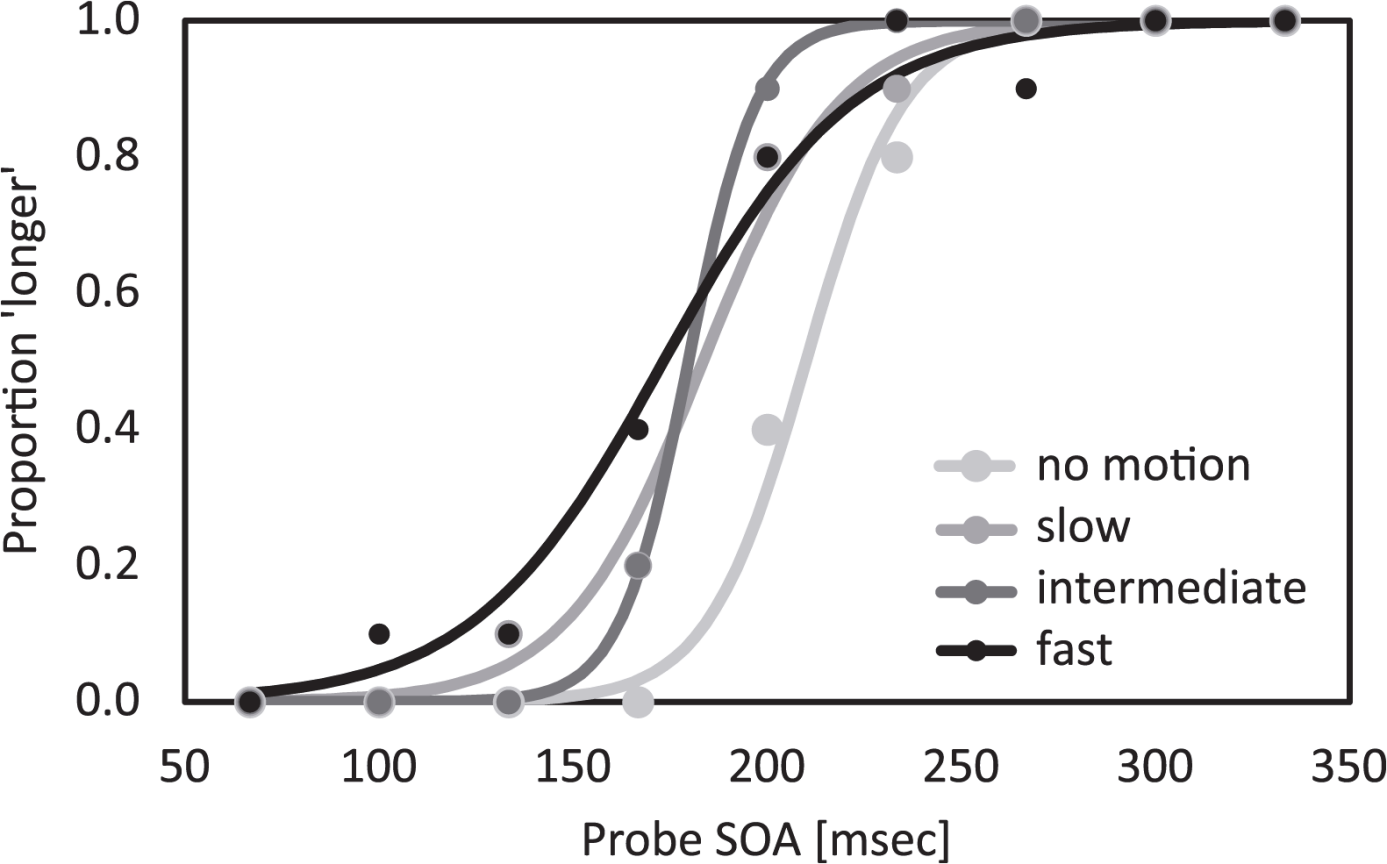
Psychometric functions for the four conditions obtained for participant T. Y. Filled circles and solid lines show the raw data and fitted psychometric curves, respectively.

Using the method of constant stimuli, we estimated the apparent test interval from the point of subjective equality (PSE; the 50% point of the cumulative logistic distribution function fitted to the psychometric function by the maximum likelihood method) and the precision of judgments from the just-noticeable difference (JND; the difference between the estimated 50% and 75% points). Examples of psychometric functions for the four conditions obtained for one participant are shown in Fig 2.

**Fig 3:**
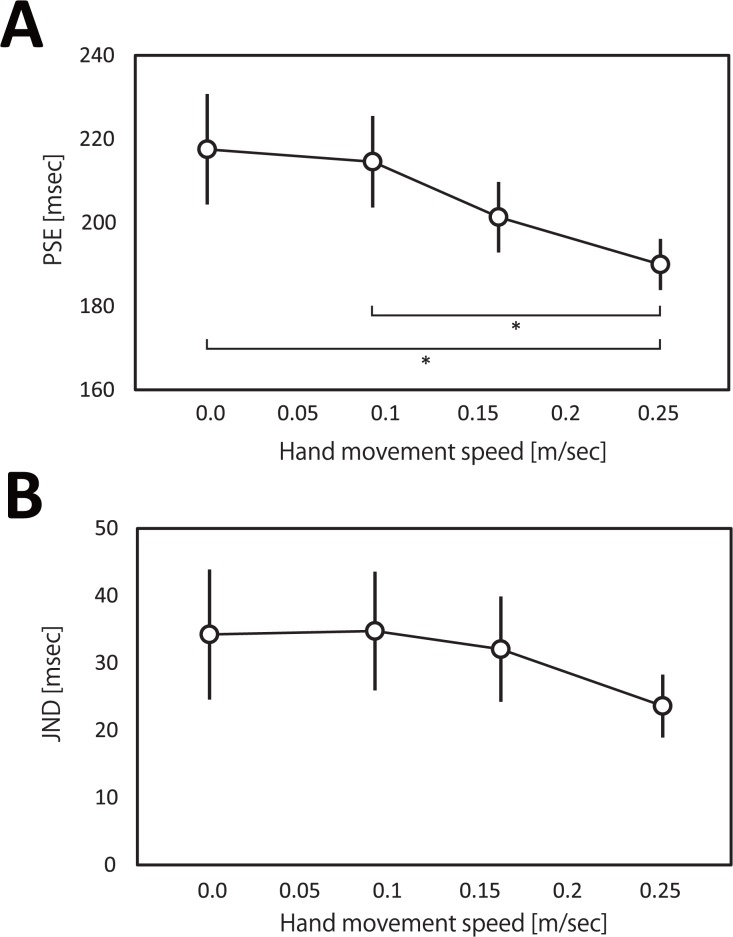
The results in the main experiment (n = 7). (A) The means of the PSEs. Error bars denote the standard errors. (B) The means of the JNDs. Error bars denote the standard errors.

The obtained PSEs are shown in Fig 3A. A one-way repeated measures ANOVA indicated a significant effect of hand movement speed (F(3, 24) = 4.23, P < 0.05, *η* ^2^ = 0.26). Ryan’s method indicated that the PSE in the fast hand movement condition was significantly smaller than that in the no-motion condition (t(18) = 3.15, P < 0.05) and slow-hand-movement condition (t(18) = 2.81, P < 0.05). These results indicate that execution of fast hand movement compressed the perceived 200 ms interval. The obtained JNDs are shown in Fig 3B. A one-way repeated measures ANOVA indicated no significant effect of hand movement speed (F(3, 24) = 1.12, P = 0.37, *η* ^2^ = 0.08). These results indicate that execution of hand movement did not affect the precision of judgments.

Since it is known that eye movements can track unseen hand movements [17–19] and that the execution of saccadic and smooth pursuit eye movements compresses subsecond durations in vision [13, 14, 20, 21], one may consider that our participants might have made saccadic or smooth pursuit eye movements during the interval estimation task even though they were instructed to look at the fixation point. To briefly examine this possibility, we conducted an experiment in which we measured participants’ eye movements during trials and calculated the magnitude of eye movements (RMSE; see Method section) in the no-motion and fast-hand-movement conditions (supplementary experiment). If the compression of the test interval in the fast-hand-movement condition resulted from participants’ eye movement [13, 14, 20, 21], the compression of the test interval in the fast-hand-movement condition should disappear when the trials in which participants moved their eye are excluded from the data analysis.

**Fig 4:**
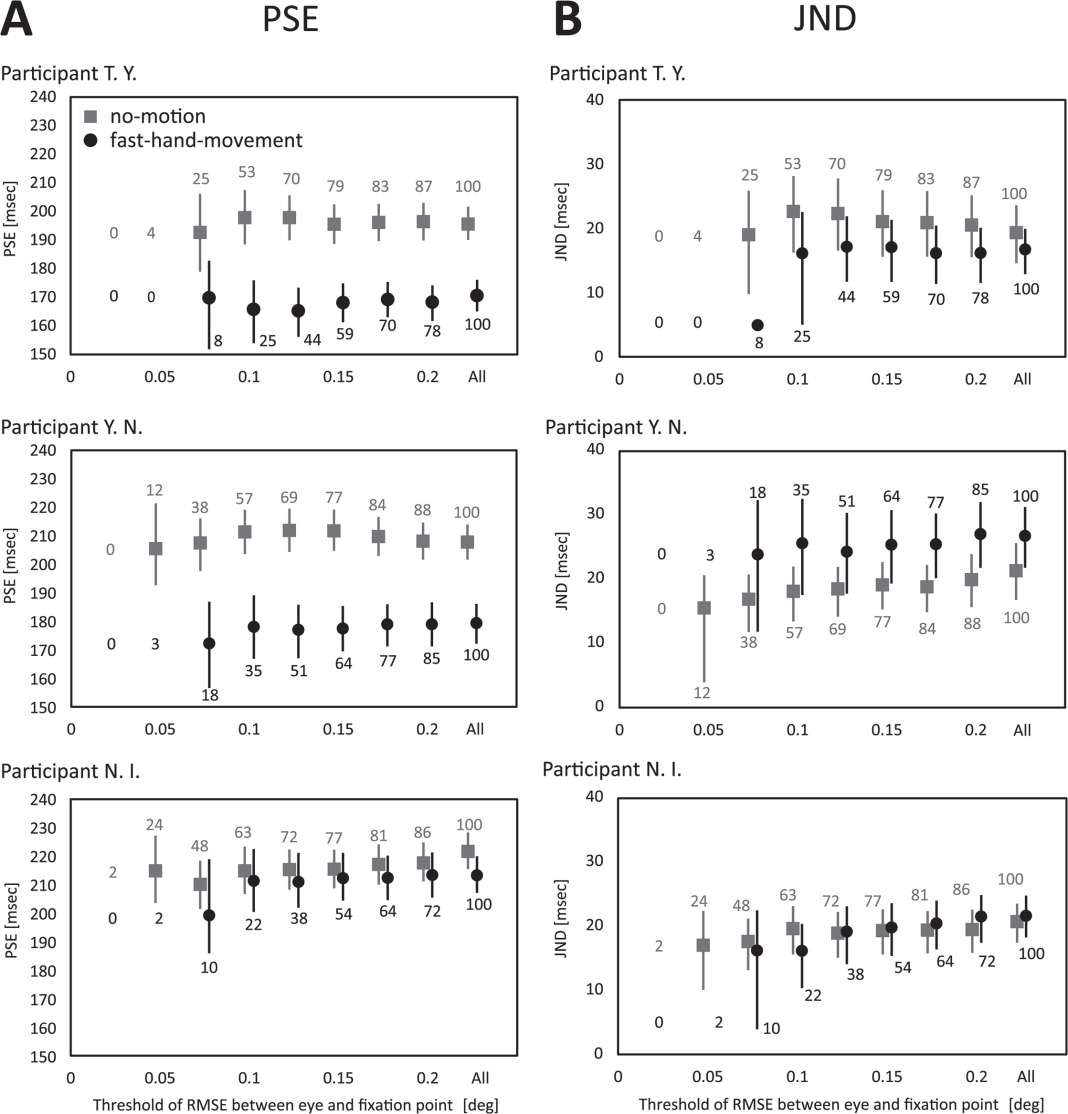
The PSEs and JNDs in the supplementary experiment (n = 3). Symbols indicate individual data points and error bars denote the 95% confidence interval calculated by bootstrap method [22]. The trials whose RMSE of eye movement exceeded the threshold were excluded from data analysis. The numbers at each data point indicate the rate of the number of sample data used to analyze all sample data (%). 100 (%) means that all 60 sample data were used in data analysis.

Fig 4 shows the PSEs and JNDs of three participants when we continuously changed the threshold of the magnitude of eye movements and calculated the psychometric function for the trials whose magnitude did not exceed the threshold. As shown in Fig 4A, the time compression effect was evident for two of the three participants. Their data showed that the PSEs were significantly smaller for the fast-hand-movement condition than for the no-motion condition even when we lowered the threshold to the utmost limit of the data sample. Furthermore, the magnitude of the time compression effect remained nearly the same even when we did not exclude the trials with fairly large eye movements. These results suggest that eye movement cannot explain our compression effect of the estimated interval. In addition, as shown in Fig 4B, the JNDs did not change across all participants.

## Discussion

The present work investigated whether the estimated interval marked by visual events is affected during the execution of hand movement. We showed that the execution of fast hand movements compressed the estimated subsecond duration of intervals marked by visual events. In addition, this compression effect could not be explained by the participants’ eye movement.

Earlier studies reported that perceived time is prolonged before and after voluntary action [12, 16]. If the estimated duration of a visual stimulus during hand movement is determined by the modality combination (hand movement and visual time), the estimated apparent interval during hand movement should be prolonged. On the other hand, it has been shown that the subsecond time of visual stimuli is compressed during the execution of a saccadic or smooth pursuit eye movement [13, 14] and that the subsecond time of tactile stimuli is also compressed during a ballistic hand movement [15]. If the estimated duration of a visual stimulus during hand movement is affected dominantly by the relative timing between stimulus and action, the estimated apparent interval presented during hand movement should be compressed. Our results showed that the apparent time interval in vision is compressed during fast hand movements as it is during eye movements [13, 14]. This suggests that the relative timing between stimulus and action is a more critical factor in determining the effect of action on time perception than the modality combination of stimulus and action.

It has been reported that the earlier temporal distortions, which would be affected by the relative timing between stimulus and action, are modality-specific (e.g., no compression of auditory time intervals during an eye movements [13,14]) or effector-specific (e.g., during hand movement, no compression of tactile time measured for the other hand [15]). However, the present temporal distortion indicates a modality-nonspecific effect between hand movement and visual time estimation. Although whether the temporal distortions found in early studies and the present ones share common mechanisms remains unknown, this is the first demonstration of effector-nonspecific temporal distortion around the time of an action.

Terao et al. reported that subsecond time intervals defined by a pair of visual flashes are apparently compressed under conditions where transient visual responses are reduced [23]. They suggested that weak transient responses fail to trigger the proper detection of temporal asynchrony, leading to increased perception of simultaneity and apparent time compression. It is also known that visual sensitivity is modulated by eye movements and that somatosensory sensitivity is modulated by a reaching movement [24, 25]. The larger the saccade is, or the faster the hand movement is, the more the sensory sensitivity is reduced [26–29]. Although the modulation effects of hand movements on visual sensitivity remain unknown, the present finding is consistent with the possibility that, following the scenario suggested by Terao et al. [23], fast hand movement weakens transient visual responses and then compresses the apparent interval. It would be interesting to investigate whether fast hand movements like we used here indeed suppress transient visual responses.

Studies of haptic localization [30–33] have reported that a stimulus presented immediately before or during hand movement is localized toward the direction of hand movement. In addition, larger mislocalization was observed during fast hand movements than during slow ones [33]. The modulation of visual temporal perception during hand movement (our results) might be similar to the modulation of haptic localization during hand movement in that both types of modulation are strengthened for fast hand movement. It is noteworthy that the haptic mislocalization can result from temporal uncertainty about the occurrence of the stimulus [33], as discussed for our results in the previous paragraph. It would be important to look into the cause of these similarities to obtain new clues about these mechanisms.

It is known that the allocation of attention to stimuli increases estimated durations [34–36] and the lack of attention to stimuli decreases them [37]. If our participants had diverted their attention from the test interval judgment to the secondary task (hand movement), particularly in the fast-hand-movement condition, the effect of attention might explain the apparent time compression [38–42]. However, although it is also known that a lack of attention to temporal stimuli decreases the precision of judgments [37–41], the precision did not change in the fast-hand-movement condition. Rather, the JND decreased with increasing hand speed as opposed to the expected effects of attention, though the decrease was not statistically significant. Therefore, attention is unlikely the main factor in the apparent time compression we observed here.

Earlier studies indicated that the apparent duration of visual input is prolonged before and after the execution of a hand movement [12, 16], and our results indicate that the apparent duration of visual input is shortened during the execution of a hand movement. Taken together, one might speculate that the compression effect during a hand movement compensates for the dilation effect before and after the hand movement in such way as to keep the total apparent time length around a hand movement veridical.

In summary, we examined whether the estimation of the temporal interval of visual events during hand movement is expanded, as in studies of visual time estimation before or after hand movement [12, 16], or compressed, as in studies of visual time estimation during eye movement [13, 14] and of tactile time estimation during hand movement [15]. The results indicate that hand movements, at least fast ones, compress the apparent time interval of a visual stimulus. This time compression cannot be explained by involuntary eye movements during the execution of hand movement. Although the present data does not allow a definite conclusion about the mechanism, our findings offer a new perspective on visual temporal interval estimation during body movement. Further studies are warranted to elucidate the mechanism underlying the sensory-motor interaction on temporal perception.

## Methods

### Participants

Six naïve volunteers (three females and three males) and one author (male) with an age range of 23–41 years participated in the main experiment. Two naïve volunteers (males) and one author (male) with an age range of 23–26 years participated in the supplementary experiment. All participants were right-handed, had normal or corrected-to-normal vision, had no known abnormalities of their motor systems, and gave written informed consent. The study’s protocol was approved by the NTT Communication Science Laboratories Research Ethics Committee and was performed in accordance with ethical standards outlined by the Declaration of Helsinki.

### Apparatus

Visual stimuli were generated by a computer and presented on a computer screen (DELL, U2312HM, 1920 x 1080, 60 Hz). The viewing distance to the monitor was 500 mm and the monitor’s field of view was 32 degrees horizontally and 54 degrees vertically. Participants sat at a table and looked down at the screen reflected in the mirror. Live video images of the participant’s moving hand were captured with a camera (Point Grey Research, Firefly MV, 752 x 480, 60 Hz) and observed only by the experimenter. The location and angle of the camera were such that real-time images of the participant’s hand movements reflected in the mirror appeared in the same spatial location as the actual movements and appeared from the same perspective as that of the hand’s being viewed directly. Captured images could be displayed raw or manipulated. The index finger position during hand movement was measured with a Kinect sensor (Microsoft Corporation, 30 Hz). Participants’ surrounding view was blocked by covering the experimental system with a blackout curtain. In the supplementary experiment, the movements of the eye were monitored at 500 Hz with an eye movement measurement system (SR Research Ltd., EyeLink II).

### Procedure

#### Main experiment

A timeline chart of the experiment is shown in Fig 1B. Each trial started with the presentation of a uniform gray field and a black fixation point (0.11 deg) at the center of the screen. After a 1-s delay, a white background image was presented, and participants started to move their right hand at any speed of the four speed conditions: 0.000 (no motion), 0.087 (slow), 0.164 (intermediate), 0.251 (fast) m/s. Although participants could not observe their hand, the experimenter observed it with the camera and compared participants’ hand movements to his own prerecorded hand movement. The speed of the experimenter’s hand movement in the prerecorded video was 0.101 m/s. By modulating the playback speed of this prerecorded video, the experimenter could create any one of the speed conditions above. The experimenter instructed participants to increase or decrease their hand speed to equalize the participants’ and prerecorded hand movement speeds. When experimenter judged that participants’ and prerecorded hand speeds were equal, a trial was started. Participants were not asked to compare their own and the prerecorded hand speeds directly in order to rule out the possibility that visual information about these hand movements could affect to the estimation of time [2–6, 43, 44]. After a random delay (5–6 s), a pair of black flashes was presented (test interval). Both flashes were disks (diameter: 1.72 deg) and appeared on the fixation point. Each flash was presented for 33 ms. The onset-to-onset flash interval was fixed at 200 ms. The participants were instructed to stop their hand movement immediately after the test interval had been presented. Two seconds later, another pair of black flashes was presented (probe interval). The onset-to-onset flash interval was randomly chosen from 67, 100, 133, 167, 200, 233, 267, 300, and 333 ms (horizontal axis in Fig 2). After an additional 500-ms blank, a uniform gray field was presented and participants reported which interval appeared longer by pressing one of two keys. We instructed participants to judge the interval between the two flash onsets (i.e., stimulus-onset asynchrony). Participants performed ten trials in each of the four movement conditions and nine SOA conditions.

#### Supplementary experiment

The experimental procedure was the same as in the main experiment except that only the no-motion and fast-hand-movement (0.251 m/s) conditions were used and the RMSE (root mean square error) of the positions of the eyes and fixation point were calculated to exclude the eye movement factor. The RMSEs were calculated among a 300-ms interval from 33.3 ms before until 33.3 ms after the test interval onset presentation period [45]. Participants performed 60 trials for each of the nine SOAs of two conditions.

### Data analysis

Each psychometric function was fitted by a cumulative logistic distribution function to the data by the maximum likelihood method to estimate the 50% correct point (i.e., PSE) and the difference between the estimated 50% and 75% points (i.e., JND). In order to calculate the radii of the hand movements, an ellipse function was fitted to the hand movements data. The mean of the major axis and minor axis of the fitted ellipse was used as the radius of the hand movements.
